# A Case Report of Takotsubo Cardiomyopathy With Dengue

**DOI:** 10.7759/cureus.42774

**Published:** 2023-07-31

**Authors:** K. V. P. Munasinghe, F. H. D. S. Silva

**Affiliations:** 1 General Medicine, Colombo South Teaching Hospital, Colombo, LKA; 2 Medicine, Faculty of Medical Sciences, University of Sri Jayewardenepura, Colombo, LKA

**Keywords:** acute coronary syndrome, takotsubo cardiomyopathy, catecholamine, apical dyskinesia, dengue

## Abstract

A 74-year-old woman with well-controlled hypertension and dyslipidemia with acute fever was diagnosed with dengue infection. She had non-anginal central chest pain which was associated with ST elevation and T inversions in V1 to V6 with prolonged QT interval. Her high-sensitivity troponin was elevated. There was echocardiographic evidence of severe left ventricular dysfunction (ejection fraction 35%; Simpson method) with apical ballooning suggestive of takotsubo cardiomyopathy. No left ventricular basal hyperkinesia was noted. The patient was managed as per the national dengue guidelines of Sri Lanka. Her cardiac condition was managed conservatively. She did not experience dengue complications such as dengue shock syndrome or dengue hemorrhagic fever or pulmonary edema secondary to severe LV dysfunction. The clinical symptoms and echocardiographic findings of takotsubo cardiomyopathy resolved parallel to dengue fever.

## Introduction

Takotsubo cardiomyopathy also known as stress cardiomyopathy, apical ballooning syndrome, or “broken heart syndrome” is a syndrome characterized by transient left ventricular dysfunction with apical ballooning [1]. Though it is a diagnosis of exclusion, co-existing occlusive coronaries in the angiogram or acute rupture of an atherosclerotic plaque cannot completely rule out the diagnosis. Postulated pathogenic mechanisms include catecholamine excess, coronary artery spasm, and microvascular dysfunction [2]. Concerning the available literature many cases of takotsubo cardiomyopathy have been reported in complicated dengue fever such as in dengue hemorrhagic fever or dengue shock syndrome rather than in simple viral infection. Hereby, we report an atypical presentation of uncomplicated dengue fever complicated with takotsubo cardiomyopathy.

## Case presentation

A 74-year-old woman with well-controlled hypertension and dyslipidemia presented with a four-day fever. It was associated with chills and rigors, headache, and retro-orbital pain with reduced urine output. She had an atypical chest pain described as a burning sensation for two days duration. The patient did not have any shortness of breath or palpitations.

At presentation, she was hemodynamically stable with a supine blood pressure of 120/70 mmHg with no postural hypotension with a pulse rate of 64 beats per minute. The oxygen saturation was 99% on air and the respiratory system examination was normal. The cardiac examination revealed a dual rhythm with no murmurs.

There was leucopenia with neutrophil predominance along with thrombocytopenia on admission. The platelet count was 80 x 10^9^/L on admission with a 29 x 10^9^/L as the lowest. She showed positivity to dengue nonstructural protein antigen 1 (NS 1) on day 2 of her illness. Dengue immunoglobulin M (IgM) was positive on the fifth day of the illness. The aspartate transaminase (AST) was 124 U/L and alanine transaminase (ALT) was 41 U/L with preserved renal functions with a normal serum creatinine and a CRP of 15 mg/L (Table 1).

**Table 1:** Hematological and biochemical parameters

Day of illness	Reference range	D3		D4		D5		D6	
Day of admission		D1		D2		D3		D4	
White blood count (x10^9^/L)	4-10 x 10^9^/L	3.5	3.4	4.3	4.4	4.5	5.2	5.6	5.8
Haemoglobin g/dL	12-16g/dL	14.7	13.8	13.5	13.5	13.4	13.6	13.4	13.2
Packed Cell Volume	37%-54%	38		39		37		38	
Platelet count (x10^9^/L)	150-400 x10^9^/L	80	57	41	29	38	52	54	61
AST U/L	<35 U/L	124						100	
ALT U/L	<35 U/L	59						41	
Creatinine µmol/L	74-100 µmol/L	76						78	
CRP mg/L	<6 mg/dL	15						8	
RBS mg/dl	<140 mg/dL	141							
Troponin I ng/L	< 30 ng/L	16.5 x 10^3^						9.6 x 10^3^	
Dengue IgM						Positive			
Dengue IgG						Negative			

**Figure 1:**
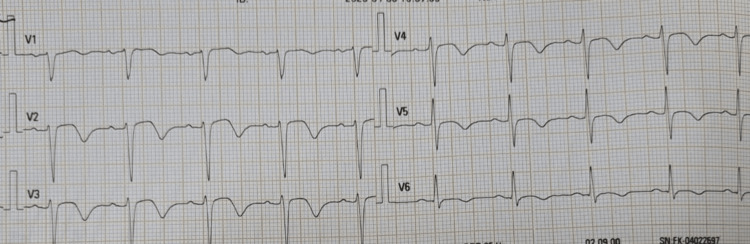
Electrocardiogram (ECG) on admission showing ST elevation and T inversions in V1 to V6 with prolonged QT interval

**Figure 2:**
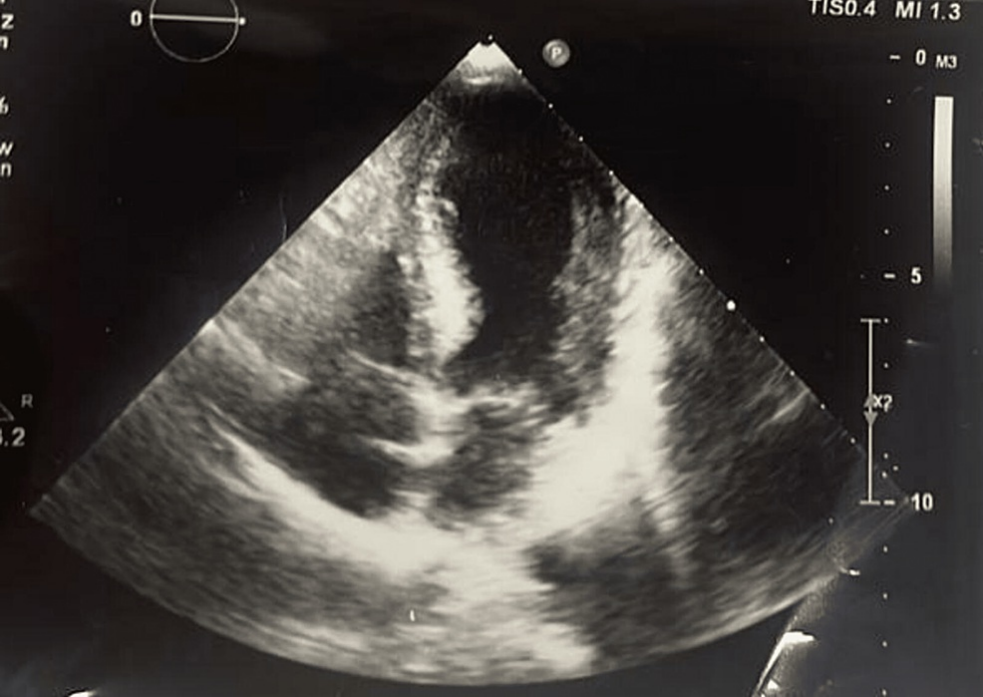
Transthoracic echocardiography showing apical ballooning

Her electrocardiogram (ECG) on admission revealed ST elevation and T inversions inV1 to V6 with prolonged QT interval (Figure 1). A high-sensitivity troponin assay showed 16.5 x 10^3 ^ng/L. Her transthoracic echocardiogram (TTE) revealed severe left ventricular systolic dysfunction with a reduced ejection fraction of 35% and apical dyskinesia suggestive of takotsubo cardiomyopathy (Figure 2).

The patient was managed as dengue fever as per national dengue guidelines with meticulous monitoring and careful oral and intravenous fluid delivery to prevent pulmonary edema [3]. She did not develop the fluid leakage phase of dengue as confirmed by repeated ultrasonography with absent gallbladder wall thickening, pleural effusions, or ascites. Dual antiplatelet agents and low molecular weight heparin were not administered due to thrombocytopenia. Once the platelet count was greater than 50 x 10^9^/L and 100 x 10^9^/L, aspirin and clopidogrel and enoxaparin were commenced respectively with careful observation for the development of bleeding manifestations.

**Figure 3:**
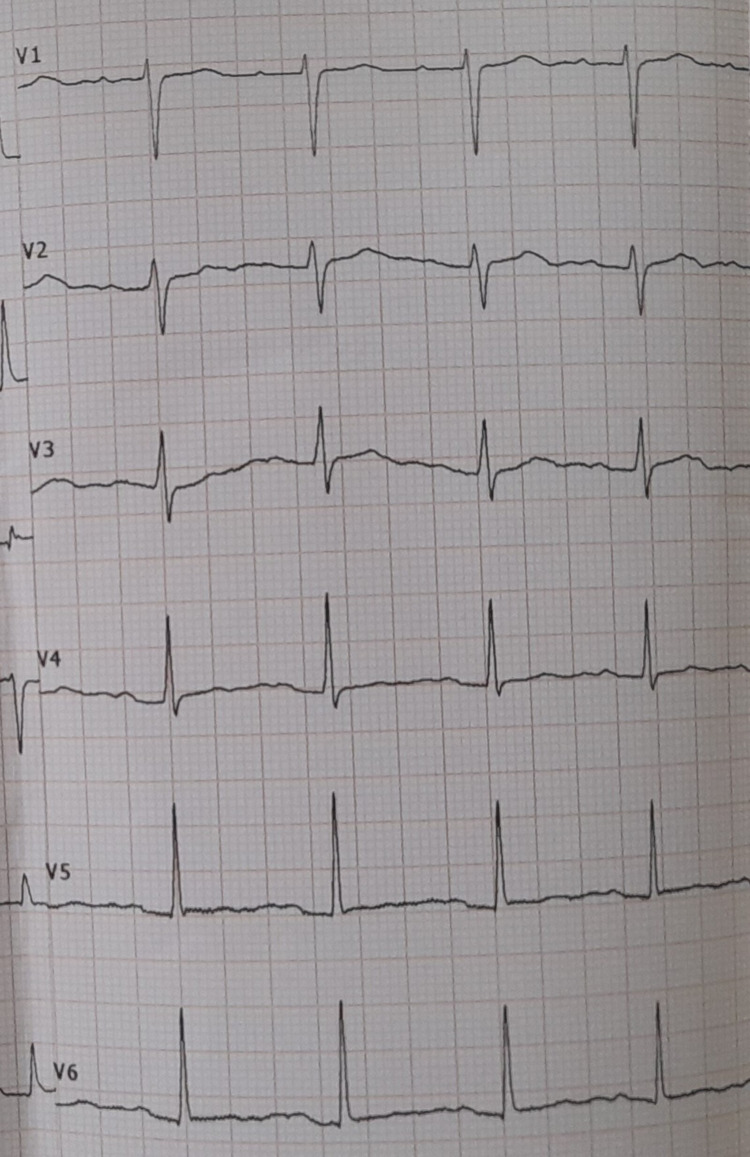
ECG after a week with complete resolution

The patient did not develop overt cardiac failure or arrhythmia and improved clinically. Recovery was evidenced by the rising platelet counts and resolution of high troponin I. She was discharged from the hospital with a plan to review as an outpatient. The follow-up TTE in three weeks revealed no regional wall motion abnormalities with an improved ejection fraction of 60% and the ECG which was repeated in one week showed complete resolution (Figure 3). An elective coronary angiogram, which was done one week after complete recovery from dengue fever did not show significant plaque formation or occlusions. After excluding underlying acute coronary syndrome, which was further reinforced by the negative coronary angiogram, anti-platelet and anti-coagulants were omitted.

## Discussion

Takotsubo cardiomyopathy which is first described in 1990 is derived from the Japanese term “octopus trap” [1]. This form of cardiomyopathy is more common in the elderly population with a post-menopausal female preponderance [4].

Considering the pathogenesis, several hypotheses have been postulated. Catecholamine excess, microvascular dysfunction, and coronary artery spasm are the principal ones [5]. It is suggested that diffuse catecholamine-induced microvascular spasm gives rise to the myocardial stunning or direct toxic effect of catecholamines leading to left ventricular systolic dysfunction. Furthermore, the proposed hypothesis is supported by the occasional finding of multifocal coronary spasms on coronary angiography and transient myocardial perfusion abnormalities that resolve with improvement in myopathy [2].

This patient had well-controlled hypertension and dyslipidemia in the background with no previous cardiac events with a normal baseline ECG. The ECG after hospital admission showed T-wave inversions in the anterolateral territories of the heart. From the available limited literature, the probable explanation for Takotsubo cardiomyopathy in dengue viral infection is described by the increased vascular permeability and thrombocytopenia with the presence of cerebral edema leading to the intense activation of autonomic sympathetic association causing myocardial stunning [5,6].

The clinical picture of Takotsubo cardiomyopathy is similar to that of acute coronary syndrome. They commonly present with chest pain, dyspnea, and syncope and less commonly, signs of heart failure, tachy/bradyarrhythmias, cardiogenic shock, mitral regurgitation, and sudden cardiac arrest [7]. Transient ischemic attacks or strokes are likely to develop due to embolization from apical thrombus [1]. ECG abnormalities show ST-segment elevation, depression, QT prolongation, T inversion, and nonspecific changes as well. Cardiac biomarkers and natriuretic peptides will be elevated. transthoracic echocardiography shows LV dysfunction revealing regional wall motion abnormalities commonly apical variant, left ventricular outflow tract obstruction, mitral regurgitation, and right ventricular involvement [8,9].

In this scenario, since there is an association with a viral illness, myocarditis is the other possibility. Although the clinical history, ECG changes, and hypertropninemia direct toward myocarditis, the typical apical ballooning appearance in the echocardiogram and rapid resolution of electrocardiographic changes and rapid recovery of the ejection fraction are the facts that make takotsubo cardiomyopathy more favorable. In the ideal setting, Cardiac magnetic resonance imaging (MRI) would have been the best option to differentiate between myocarditis and stress cardiomyopathy which would show myocardial edema and late gadolinium enhancement [10]. But lack of availability and affordability acted as a main constraint in this study.

Diagnosis is confirmed by transient left ventricular systolic dysfunction preceding emotional, physical, or combined trigger, new ECG changes/modest elevation of cardiac troponins, and absence of pheochromocytoma or myocarditis. It is noteworthy that nonocclusive coronaries are not always present [10].

Management of stress cardiomyopathy is supportive and if the patient develops acute complications such as heart failure, cardiogenic shock, or thrombo-embolic stroke, these need to be managed accordingly [11]. Patients who sustain an episode of stress cardiomyopathy are at risk of recurrence of 1%-2% per year [12]. Dengue is a mild illness at the initial presentation but may progress into a severe disease, it is important to foreground that dengue can manifest as various atypical presentations.

## Conclusions

Takotsubo cardiomyopathy is an atypical complication of dengue which clinicians should be mindful of. Differentiation between takotsubo cardiomyopathy, myocarditis, and acute coronary syndrome could be misleading. Careful monitoring on the prevention of fluid imbalance, shock, and avoidance of overt bleeding manifestations is crucial in the management of patients presenting with this.
